# Water, Sanitation, and Hygiene (WASH): A Critical Component for Sustainable Soil-Transmitted Helminth and Schistosomiasis Control

**DOI:** 10.1371/journal.pntd.0002651

**Published:** 2014-04-10

**Authors:** Suzy J. Campbell, Georgia B. Savage, Darren J. Gray, Jo-An M. Atkinson, Ricardo J. Soares Magalhães, Susana V. Nery, James S. McCarthy, Yael Velleman, James H. Wicken, Rebecca J. Traub, Gail M. Williams, Ross M. Andrews, Archie C. A. Clements

**Affiliations:** 1 School of Population Health, University of Queensland, Brisbane, Queensland, Australia; 2 WaterAid Australia, Melbourne, Victoria, Australia; 3 Molecular Parasitology Laboratory, QIMR Berghofer Medical Research Institute, Brisbane, Queensland, Australia; 4 Clinical Tropical Medicine Laboratory, QIMR Berghofer Medical Research Institute, Brisbane, Queensland, Australia; 5 Department of Infectious Diseases, Royal Women's and Children's Hospital, Brisbane, Queensland, Australia; 6 WaterAid, London, United Kingdom; 7 School of Veterinary Sciences, University of Queensland, Gatton, Queensland, Australia; 8 Menzies School of Health Research, Charles Darwin University, Darwin, Northern Territory, Australia; National Institute of Parasitic Diseases China CDC, China

## Introduction

Soil-transmitted helminths (STH) and schistosomes are parasites that affect the world's poorest people, causing losses of up to 39 million and 70 million disability adjusted life years (DALYs) respectively [1], [2]. The World Health Organization (WHO) is at the forefront of developing policy for the control of STH and schistosomiasis, advocating for chemotherapy as the cornerstone of control, with the objective of reducing infection-associated morbidity [1], [3], [4]. Global uptake of chemotherapy with albendazole or mebendazole for STH and praziquantel for schistosomiasis has significantly increased and remains the principal control strategy. It is cost-effective [5] and reduces STH [6] and schistosome [7] infections in human hosts.

However, a fundamental limitation of chemotherapy for STH and schistosomiasis control is that it does not kill immature worms and cannot prevent reinfection. Chemotherapy-based control programmes have a temporary effect on transmission [8]. Indeed, studies have shown that infection prevalence and intensity can rapidly return to baseline levels soon after chemotherapy programmes are ceased. One factor is that the ability of helminth eggs and/or larvae to survive for extended periods in the environment [9] creates a source for rapid reinfection following chemotherapy [9]. A second is that small sections of the population usually remain out of reach of chemotherapy programmes, subgroups that frequently have a disproportionately heavy burden of infection, thereby serving as a reservoir for reinfection. Thus, longer-term effectiveness of chemotherapy in interrupting transmission is dependent on maintenance of regular retreatment. Many helminth control programmes rely on donated drugs [3], so there is a degree of uncertainty around their sustainability in the long term. In endemic areas, once mass treatment is stopped, disease prevalence can return to pretreatment levels within 18–24 months [10]–[12]. For schistosomiasis, cessation of chemotherapy can also result in more severe rebound of immunopathology [13].

The most frequently used chemotherapeutic drug, albendazole, does not have 100% efficacy [14]; therefore, chemotherapy programmes will not cure all treated individuals. Additionally, helminth control programmes have predominantly focused on specific risk groups (primarily schoolchildren) rather than the whole community, despite evidence in many communities that prevalence may be high in other groups [15], for example, preschool children [16]. A shift in approach to community-wide chemotherapy, or at least to include preschoolers as a target population, could potentially have a great impact on further reducing STH infections, particularly in settings where there is high prevalence in nonschool groups or where many children do not go to school.

Even where there are continuous control programmes, there is some evidence of declining uptake due to fear of treatment and poor communication about the chemotherapy process [17]. There is also the potential that mass drug administration may result in drug resistance, as is occurring in livestock helminth control programmes [18]–[20]. Humphries et al. (2011) believe that, given the current treatment pressure, it will only be a matter of time before drug resistance is seen in STH species that infect humans [21]. Controversially, recent reviews indicate that, on the basis of measures of infection-associated morbidity (such as improvements in nutrition, haemoglobin levels, school attendance, and school performance), there is insufficient reliable evidence to justify contemporary chemotherapy programmes [22], [23]. We do, however, recognise that in developing country settings, where multiple disease and health-related interactions are likely to take place, it is difficult to associate nonspecific morbidity indicators to STH or schistosomiasis. Other issues that are not yet resolved with regards to chemotherapy include potential teratogenic effects of benzimidazole drugs and associations with eczema in children following maternal chemotherapy during pregnancy [24]. Thus, whilst chemotherapy is necessary to rapidly reduce the burden and morbidity of helminth infections, we argue that by itself it is an unsustainable strategy for helminth control and for reaching control and elimination targets. This highlights the essential role of interventions aimed at reducing environmental exposure, which chemotherapy alone does not address.

The provision of access to WASH, being a safe water supply, appropriately constructed sanitation infrastructure that ensures safe disposal of human excreta, and the promotion of hygiene (defined as personal and household practices such as hand-washing, bathing, and management of stored water in the home, all aimed at preserving cleanliness and health), is critical. WASH is a necessary but undervalued tool for helminth prevention and control, aiming to provide long-term improvements in people's wellbeing. Interventions that include WASH have been shown to be highly effective in reducing the environmental exposure to, and transmission of, eggs and larvae for STH [25] and schistosomes [26]. A 29% decrease in *Ascaris lumbricoides* prevalence and as much as a 77% reduction in schistosomiasis prevalence has been observed following implementation of improved water and/or sanitation facilities [25]. A recent study in three African countries estimated that the population attributable fraction (PAF) of schistosomiasis due to no piped water was 47–71% [27].

**Figure 1:**
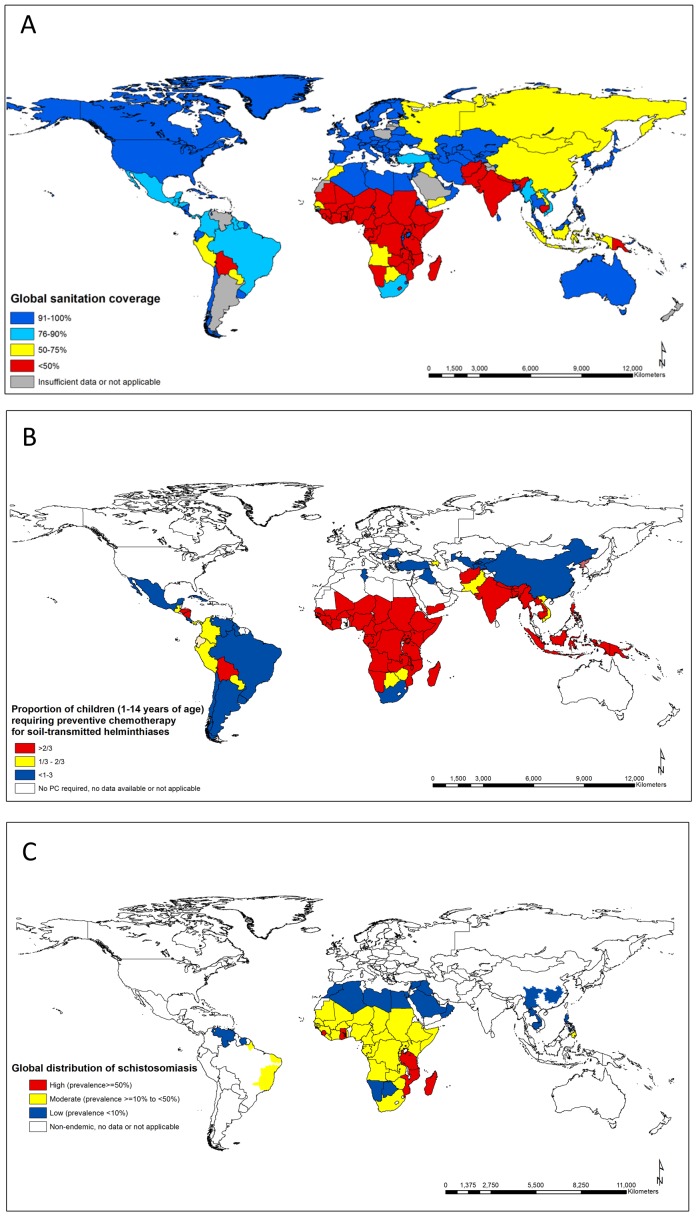
Consistencies in the global need for improved WASH and parasite control. (a) Global sanitation coverage (adapted from [51]). (b) Global requirements for chemotherapy for STH (adapted from [3]). (c) Global distribution of schistosomiasis (adapted from [3]).

Areas with poor sanitation coverage often experience a high burden of disease from STH and schistosomiasis (Figure 1). WASH implementation can be complex and comprised of a large set of “hardware” (e.g., toilets, latrines, sewage treatments, and provision of safe water) [16] and “software” (e.g., behaviour change promotion and community resource management) elements, many of which are, strictly speaking, outside the official service delivery remit of the health system. Challenges for implementing WASH can include cost, lack of health professional involvement [28], lack of local government involvement and local public-private partnerships for latrine and infrastructure development [29], lack of advocacy [30], inappropriate choice of technology, poor operation and maintenance, inadequate revenue collection, lack of adequate and equitable financial investment from both government and international donors [31], and the lack of perception in many rural communities of the importance of improved excreta disposal practices [32]. This requires genuine cross-sectoral collaboration and political will; investment in WASH in developing countries contributes to practically all of the Millennium Development Goals (MDGs) [28], and should not be overlooked for helminth control simply because chemotherapeutic interventions exist that require a seemingly lower financial and logistical commitment.

## Helminth Control Guidelines and the Neglect of WASH

For many years, authors [6], [8] have argued that the effects of chemotherapy can only be sustainable if integrated with improvements in health promotion, hygiene, and sanitation. This has been recognised and advocated for in the World Health Assembly (WHA) resolutions on STH and schistosomiasis, as well as the recent resolution on NTDs. These foundational policy guidelines clearly highlight the importance of WASH as a fundamental component of helminth control and elimination [33]–[35]; however, as discussed below, WASH is not embraced in subsequent disease-specific control guidelines (e.g., STH and schistosomiasis). A longer-term view of effectiveness and sustainability of control efforts requires integrating interventions to reduce transmission and reinfection. Yet interventions such as WASH have been slow to be incorporated into control programmes. It is for this reason that the parties to the London Declaration on NTDs are seeking more coordinated access to clean water and basic sanitation, improved living conditions, vector control, health education, and stronger health systems in endemic areas [36].

The WHO published guidelines for the prevention and control of STH and schistosomiasis infections in 2002 [1] and recently produced updated guidelines entitled “Helminth control in school-age children: a guide for managers of control programmes, 2nd edition” [3], specifically targeting STH and schistosomiasis. This second document acknowledges the importance of WASH and provides advice that helminth control programmes need to comprehensively include WASH, with the definitive statement, “The only definitive solution for eliminating schistosomiasis and STH infections is improvement in environmental conditions and a change in risk behaviours” [3]. However, chemotherapy is prioritised as the “first-line rapid control measure,” while improved water and sanitation and health education should be only “implemented according to the epidemiological situation and the availability of resources” [3]. No clear definition of what is meant by “epidemiological situation” in this context is provided. Our concern is that these last two statements will have the unintended effect of delaying action on WASH in favour of chemotherapy, without interrupting the vicious cycle of disease transmission. The guidelines could be enhanced by inclusion of comprehensive recommendations for implementing WASH hardware and software, citing methods and examples such as the Community-Led Total Sanitation (CLTS) approach, which has now been successfully implemented in over 20 countries [37], sanitation marketing, and other approaches that focus on creating demand for sanitation and changing unhealthy behaviours.

Of significant concern regarding the current WHO guidelines is that they contain no recommended control activities where prevalence of STH infection below 20% is identified at baseline [3]. Instead, following the chemotherapy focus of the document, “Affected individuals should be treated [for STH] on a case-by-case basis” (Table 2.3 in [3]); however, no suggestions for identifying these individuals are proposed. Such an approach needs to be supported by rigorous epidemiological evidence that clearly demonstrates benefits to the community concerned and appropriate mitigation of the risk of cross-infection into uninfected individuals. STH and schistosomes are extremely difficult to eliminate in communities where poverty and inadequate water and sanitation prevail, due to their high transmission potential [38]. Lack of specifying control activities in this scenario represents, at the very least, a missed opportunity for recommending WASH activities, particularly given the level of morbidity likely to be experienced in a community with 20% STH prevalence.

An additional area of the WHO guidelines that warrants close scrutiny are decision trees in the annexes, which recommend reducing frequency of chemotherapy after five to six years, based solely on measurements of prevalence. For prevalence of STH or schistosomiasis below 1%, the WHO guidelines indicate, “morbidity is under control with low risk of re-emergence,” although serology for schistosomiasis is recommended with positive cases continuing to receive chemotherapy [3]. It is unclear whether serology is intended for all schoolchildren in this scenario, and additionally there is no evidence to indicate that risk of re-emergence of disease is not a problem at this threshold level, particularly if WASH is not adequate. We propose that WASH indicators be added to the decision trees, to provide sounder guidance for programme managers in their decision-making about helminth control programmes. It would also more comprehensively mitigate risk of resurgence of STH and schistosomes, as it would address necessary environmental improvements for control, as well as demonstrate longer-term, sustainable benefits to the communities concerned.

The WHO guidelines published in 2002 [1] were the first such document of its kind. It admirably articulated a large volume of technical information to assist programme managers develop prevention and control strategies. The more recent version, however, does not seem to have progressed considerably from the earlier version. Rather, the recognition in the 2002 version that resources must not be diverted prematurely in countries where morbidity has been significantly reduced but transmission continues [1] mitigates risk more appropriately than the current second edition guidelines. We believe there is a strong justification for a further revision to be undertaken.

## Getting the Indicators Right

The current WHO guidelines use prevalence of infection as the most emphasised indicator of the success of worm control programmes, whilst the “condition of latrines and the quality of water supplies in schools may also be monitored if their improvement is one of the objectives of the programme” [3]. Use of prevalence is insufficient as it does not place emphasis on using interventions that have a more sustainable impact. Given the reinfection rate of STH and schistosomes, being guided by prevalence rates alone is high risk. As the WHO guidelines correctly point out, remaining “parasites maintain transmission capacity despite intense drug pressure, and this is predictive of a rapid return to high levels of prevalence if the [chemotherapy] intervention is interrupted” [3]. Intensity of infection (as measured by number of eggs in stool/urine) is markedly different within various groups of the community, such as different age groups and sex [39]. Thus, prevalence can easily mask the high transmission potential of a relatively small number of individuals. Hygiene activities are included with indicators for monitoring numbers of hygiene education programmes conducted, although these would not sufficiently measure hygiene behavioural change.

We recommend that, at the very least, corresponding WASH access indicators be included in any revised versions of WHO helminth control guidelines. These could include the MDG seven indicators of (i) proportion of the population using an improved drinking water source and (ii) proportion of the population using an improved sanitation facility [40], with “improved” water and sanitation defined by the WHO-UNICEF Joint Monitoring Programme for Water Supply and Sanitation [41]. These are the most developed and consistently used WASH indicators. Many national health surveys are collecting data on some of these indicators; thus the addition of these indicators should not involve adding completely novel indicators into helminth control programmes. We acknowledge that there has been some criticism of the MDG indicators with regards to equity, specifically, that the MDGs target the richer proportions of each country's population, rather than those at greatest need. This has not been resolved, and there has been a general call to develop more equitable indicators beyond 2015 [42]. However, based on current approaches, these indicators appear the most suitable at this time for ensuring that WASH is addressed in conjunction with chemotherapy.

There should also be guidance on appropriate implementation provided in the second edition WHO guidelines. Such guidance should encourage best-practice sanitation and hygiene promotion approaches relevant to the context in the programme location. The CLTS approach, which avoids the use of hardware subsidies and “latrinification” (construction of latrines for households without commensurate efforts to ensure safe sanitary practices and ownership and adequate maintenance of latrines) is one potential approach, alongside other emerging approaches such as sanitation marketing, which focuses on creation of demand for household investment sanitation hardware in order to allow progressive improvement away from basic latrines. Guidance should also specifically encourage improved coordination and planning across sectors, such as the participation of WASH agencies in national NTD task forces. It is known that sanitation does not become effective until it is used by a high percentage of the population [25], [43], with coverage of properly built, used, and maintained sanitation required to be 90% to have an effect on STH transmission [44]. If insufficient proportions of people in a community have access to sanitation, even those who have latrines will still be at risk of infection [45], particularly if there is latrine access at local schools or institutions but not within the community, or vice versa. For this reason, we advocate for universal access to WASH to be considered in MDG planning beyond 2015. In the interim, setting WASH access indicators in any revised version of WHO helminth control guidelines is a crucial next step that will help to tackle the disease burden caused by STH and schistosomiasis. An additional and significant benefit of high community WASH access would be its impact on controlling other excreta-borne pathogens, including viruses, bacteria, and protozoa [46].

There is very little literature that indicates direct WASH impact on helminth control. We believe there is an urgent need to conduct epidemiological research, including appropriately structured intervention trials [47] and mathematical modelling studies [48], [49], to evaluate the effect of integrated interventions on helminth infections and infection-associated morbidity. Existing evidence is already strong enough to support complementing drug-based interventions with the provision of WASH for all [50], but more work can be done to determine intervention thresholds for the selected WASH indicators to be incorporated into decision trees such as those presented in the annexes of the WHO guidelines.

## Conclusion

Progress towards achieving global control of helminths crucially depends on sustainable solutions that move beyond treating symptoms towards reducing exposure. With that in mind, it is necessary to augment chemotherapy with WASH and other interventions such as health promotion to achieve a cumulative impact of preventing reinfection and providing the greatest and most sustainable gains for helminth control and elimination. We believe that a strong justification exists to revise the WHO guidelines in the face of the abovementioned shortcomings. Such revision will result in a much-enhanced document that covers the full spectrum of short- and longer-term interventions for more holistic STH and schistosomiasis control. Impact indicators for WASH, in addition to disease-related indicators such as prevalence of infection, should define the success of a control programme and guide decisions as to when such programmes should cease. This would ensure current gains in helminth control are built upon beyond the current dependence on chemotherapy.
